# Genetic Diversity and Virulence Profile of Methicillin and Inducible Clindamycin-Resistant *Staphylococcus aureus* Isolates in Western Algeria

**DOI:** 10.3390/antibiotics11070971

**Published:** 2022-07-19

**Authors:** Zahoua Mentfakh Laceb, Seydina M. Diene, Rym Lalaoui, Mabrouk Kihal, Fella Hamaidi Chergui, Jean-Marc Rolain, Linda Hadjadj

**Affiliations:** 1 Laboratoire de Biotechnologies, Environnement et Santé, Faculté des Sciences de la Nature et de la Vie, Université de Blida 01, BP270 Route Soumaa, Blida 09000, Algeria; zahoualaceb@gmail.com (Z.M.L.); hamaidifella@yahoo.fr (F.H.C.); 2 Faculté de Médecine et de Pharmacie, IRD, APHM, MEPHI, Aix Marseille University, 19-21 Boulevard Jean Moulin, CEDEX 05, 13385 Marseille, France; seydina.m.ddiene@gmail.com (S.M.D.); l.rym-microbio@hotmail.fr (R.L.); jean-marc.rolain@univ-amu.fr (J.-M.R.); 3 IHU Méditerranée Infection, 13005 Marseille, France; 4 Laboratoire de Microbiologie Appliquée, Université Ahmed Ben Bella Oran1, BP1524 El M’naouer, Oran 31000, Algeria; kihalm@gmail.com

**Keywords:** *Staphylococcus aureus*, methicillin-resistant *S. aureus* MRSA, inducible macrolide lincosamide streptogramin B (iMLSB), virulence, Algeria

## Abstract

*Staphylococcusaureus* causes a wide range of life-threatening infections. In this study, we determined its prevalence in the hospital environment and investigated nasal carriage among healthcare workers and patients admitted to a hospital in western Algeria. A total of 550 specimens were collected. An antibiogram was performed and the genes encoding resistance to methicillin, inducible clindamycin and toxins were sought among the 92 *S. aureus* isolates. The spread of clones with a methicillin- and/or clindamycin-resistance phenotype between these ecosystems was studied using genomic analysis. A prevalence of 27%, 30% and 13% of *S. aureus* (including 2.7%, 5% and 1.25% of MRSA) in patients, healthcare workers and the hospital environment were observed, respectively. The presence of the *mecA*, *erm*, *pvl* and *tsst-1* genes was detected in 10.9%, 17.4%, 7.6% and 18.5% of samples, respectively. Sequencing allowed us to identify seven sequence types, including three MRSA-IV-ST6, two MRSA-IV-ST80-PVL+, two MRSA-IV-ST22-TSST-1, two MRSA-V-ST5, and one MRSA-IV-ST398, as well as many virulence genes. Here, we reported that both the hospital environment and nasal carriage may be reservoirs contributing to the spread of the same pathogenic clone persisting over time. The circulation of different pathogenic clones of MRSA, MSSA, and iMLSB, as well as the emergence of at-risk ST398 clones should be monitored.

## 1. Introduction

*Staphylococcus aureus* has adapted to human hosts and the hospital environment and is a leading cause of nosocomial and community-acquired infections. At the same time, it is a commensal bacterium and a major cause of endocarditis, soft tissue infections, skin infections and osteomyelitis [1]. In addition to being resistant to antibiotics, its pathogenicity is also related to virulence factors including surface proteins, enzymes and toxins such as Panton Valentine Leukocidin (PVL) and Toxic Shock Syndrome Toxin-1 (TSST-1) [2]. Methicillin-resistant *S. aureus* (MRSA) is an alarming feature, which first emerged as a healthcare-associated infection (HA-MRSA) in 1960, before expanding to the community (CA-MRSA) in 1980 [3]. The genetic diversity of MRSA has led to the serial emergence of epidemic strains worldwide [1]. Multiple sites in the human body harbour *S. aureus*, including the gastrointestinal tract and the intestines, although nasal carriage remains the primary site of this colonisation [4]. It is estimated that between 15% and 36% of the world’s population is colonised by *S. aureus*. The infection of colonised patients is a significant cause of transmission, as postoperative bacteraemia resulting from intraoperative transmission associated with preoperative nasal carriage is very common [5,6,7]. The hospital environment can also be highly contaminated by *S. aureus* through its presence on surfaces and objects. One of its biological advantages is that it can survive for long periods on surfaces, which makes attempts to eradicate it difficult [8,9]. Contaminated hospital environments are, therefore, a source of cross-infection and act as a potential reservoir of nosocomial pathogens. Data are insufficient on the prevalence of nasal carriage of *S. aureus* in Africa as well as on the epidemiology and characterisation of circulating clones. The few studies that do exist are mainly from developed countries, and their infection control and surveillance practices are not applicable to some African countries [10,11]. The use of genomics is a highly efficient method of identifying and characterising *S. aureus* strains and of monitoring their progress.

The objectives of this article are first to determine the prevalence of *S. aureus* in the hospital environment as well as nasal carriage among patients and healthcare workers (HWs) in a hospital in western Algeria. We then go on to characterise the resistance and virulence phenotypes of the isolated strains and finally, determine the clones circulating in the hospital and their mode of diffusion over a period from November 2020 to May 2021.

## 2. Materials and Methods

### 2.1. Study Design

Our cross-sectional study was conducted between November 2020 and May 2021 in the orthopaedic surgery, general surgery and intensive care units of a hospital located in the west of Algeria. This hospital provides tertiary care to the population of the city and the entire western region.

A total of 550 samples were collected and analysed, of which 400 were from the hospital environment (various surfaces and biomedical equipment), 110 were from the nares of hospitalised patients within 48 h of their admission for planned surgery, and 40 were from HWs. Details on sampling dates, site and other information are available in Supplementary Tables S1–S3. 

Samples were collected using moistened sterile swabs that were wiped on different sites of frequently affected biomedical surfaces and equipment [12]. To investigate nasal carriage of *S. aureus*, we inserted swabs into both nostrils [13]. The swabs were promptly transferred to the microbiology laboratory for analysis. 

### 2.2. Microbiological Analysis

After enrichment of the samples in Brain Heart Infusion Broth (bioMérieux, Mercy l’Etoile, France) at 37 °C for 24 h, we performed an isolation on mannitol salt agar (bioMérieux, Mercy l’Etoile, France) at 37 °C for 48 h. The identification of isolated strains as *S. aureus* was also based on the DNASE test (Bio-Rad, Marnes-la-Coquette, France). This was then confirmed by matrix-assisted laser desorption ionisation/time of flight (MALDI-TOF) (Bruker, Bremen, Daltonics, Germany) [14].

### 2.3. Antimicrobial Resistance Phenotype

We investigated antibiotic susceptibility using a panel of 16 antibiotics (penicillin, cefoxitin, oxacillin, rifampicin, clindamycin, erythromycin, pristinamycin, gentamicin, vancomycin, teicoplanin, doxycycline, fosfomycin, ciprofloxacin, fusidic acid, linezolid, and trimethoprim/sulfamethoxazole) using the disc diffusion method on Muëller Hinton agar (Beckton Dickinson, Rungis, France). The results were interpreted according to EUCAST recommendations. The iMLSB phenotype for inducible clindamycin resistance was detected by D-test [15]. 

### 2.4. Screening for Resistance and Virulence Genes

DNA from all strains was extracted using a commercial DNA extraction kit: EZ1 DNA with the BioRobot EZ1 (Qiagen, Courtaboeuf, France). The detection of the *mecA*, *mecC*, *pvl,* and *tsst-1* genes was performed by RT-PCR, while the *erm(A)*, *erm(B)*, *erm(C)*, *erm(T)* and *msr(A)* genes were detected by standard PCR [16,17,18,19,20,21]. 

### 2.5. Whole Genome Sequencing (WGS)

A total of 22 strains with MRSA and/or iMLSB profiles were selected for WGS using MiSeq (Illumina Inc., San Diego, CA, USA). The quality of raw sequencing data was checked by FastQC and filtered using the fastq-mcf program [22]. The reads were then assembled using the SPAdes software (Galaxy version 3.12.0 + galaxy1) [23] and annotated with Prokka (Galaxy version 1.14.6 + galaxy1) [24]. Antimicrobial resistance and virulence genes were detected with Abricate, while the SCC*mec* type, MLST and Spa typing were determined using the various tools on the Center for Genomic Epidemiology website [25,26,27,28]. Pangenome analysis was performed using Roary software (version 3.13.0) with default parameters.

## 3. Results

### 3.1. Bacterial Isolates

A total of 92 *S. aureus* were recovered from 110 patients, 40 HWs and 400 samples from the hospital environment. Table 1 shows the prevalence of nasal carriage in patients (27%), HWs (30%) and environmental samples (13%).

Contamination was highest in wet surfaces, followed by serum racks and respirators in the intensive care unit and bed surfaces. Details of the screening conducted at the various sites are available in Supplementary Table S1.

### 3.2. Antimicrobial Susceptibility 

**Figure 1:**
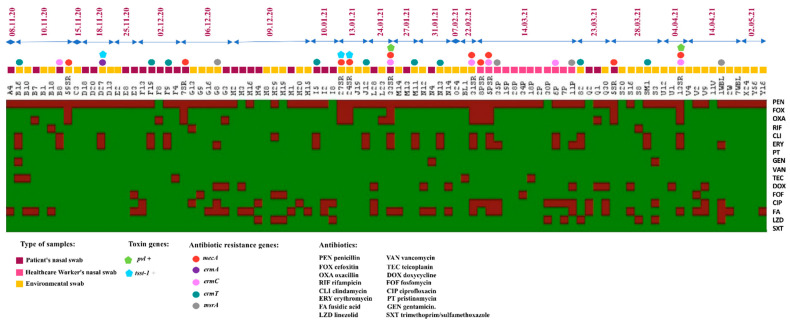
Illustration of the resistance phenotype, and distribution of resistance genes and toxins tested according to the origin of the isolates and grouped by the chronology of their isolation.

As shown in Figure 1, isolates expressed the highest level of resistance to penicillin at 87% (*n* = 80), followed by fusidic acid at 35% (*n* = 32), ciprofloxacin at 23% (*n* = 21), erythromycin at 22% (*n* = 20), clindamycin at 17% (*n* = 16), oxacillin at 16.3% (*n* = 15), and cefoxitin at 10.8% (*n* = 10). Low levels of resistance (<11%) were recorded for the other antibiotics.

The iMLSB phenotype with a positive D-test was detected in 16 strains (Figure 1).

### 3.3. Screening for Resistance and Virulence Genes

Of the 92 strains isolated, we identified 10 strains carrying the *mecA* gene and none carrying *mecC*. The macrolide-resistance genes detected were *ermT* (*n* = 9), *ermA* (*n* = 1), *ermC* (*n* = 6) and *msrA* (*n* = 4), but there were no *ermB* genes. Concerning toxins, the gene coding for PVL was found in 7 isolates and that for TSST-1 was found in 17 strains (Figure 1). 

### 3.4. Analysis of WGS

**Figure 2:**
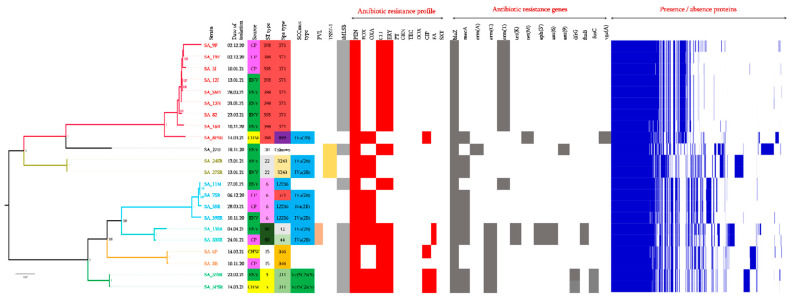
Pangenome analysis of the 22 sequenced *S. aureus* isolates, their date of isolation, the presence of PVL and TSST, their antibiotic resistance profile, and genetic features. CP: carriage patient, Env: environmental, CHW: carriage health worker, PEN: penicillin, FOX: cefoxitin, OXA: oxacillin, DOX: doxycycline, CLI: clindamycin, CIP: ciprofloxacin, ERY: erythromycin, PT: pristinamycin, FA: fusidic acid, GEN: gentamicin, TEC: teicoplanin, SXT: trimethoprim/sulfamethoxazole.

Genome analysis revealed genes encoding resistance to β-lactams (*blaZ, mecA*), tetracycline (*tetM*, *tetK*), fusidic acid (*fusB*, *fusC),* aminoglycoside (*aph3′*, *ant6-Ia*, *ant9-Ia*), sulfamides (*dfrG),* and streptogramin (*vgaA*) (Figure 2). The *mecA* gene is carried on two types of mobile genetic elements: SCC*mec* IVa (2B) and Vc (5C2&5).

Eight spa types were identified (t311; t3243; t12236; t346; t571; t042; t044; t899) and one was unknown. The strains were classified in seven different ST, mainly ST398 (*n* = 9), then ST6 (*n* = 4), ST5, ST22, ST80, ST15 (*n* = 2) and ST30 (*n* = 1).

Concerning MRSA isolates, MRSA-IV-ST6 (*n* = 3), MRSA-IV-ST80-PVL+ (*n* = 2), MRSA-IV-ST22-TSST-1+ (*n* = 2), MRSA-V-ST5 (*n* = 2) and MRSA-IV-ST398 (*n* = 1) strains were observed in the hospital environment but also in some healthy carriers and patients. The pangenome highlights the similarity between the strains from the hospital environment and those of certain patients (ST80, ST6), or from the environment and healthcare workers (ST5), despite the fact that they were collected months later. The evolution of ST398 can also be seen in Figure 2. A large number of virulence genes were detected, some of them coding for toxins, haemolysins, adhesins and capsule components (Table 2).

## 4. Discussion

In order to estimate the potential risks that *S. aureus* poses to human health and its circulation in hospital departments, it is crucial to study potential reservoirs and routes of transmission. In this study, we evaluated the prevalence, antibiotic susceptibility, virulence, and clonal diversity of MSSA/MRSA recovered from environmental surfaces, biomedical equipment, and patients as well as HWs.

In our case, the prevalence of preoperative nasal *S. aureus* carriage in patients was 27%, including 2.7% MRSA. These rates are quite similar to studies from the center and east of Algeria [3,29] and Ghana, but lower than in Senegal [30]. In Australia, the detection of MRSA is lower, at 0.7% [31].

In HWs, the prevalence of *S. aureus* was 30% with 5% MRSA. This rate comes close to the results observed in the Iranian population, which revealed 37% of *S. aureus* with 4% MRSA [32]. In the Democratic Republic of the Congo, lower rates were reported, with 16.6% *S. aureus* and 2.6% MRSA [33]. This type of carriage in humans could contribute towards the transmission of care-associated infections, either as vectors or reservoirs [34]. Systematic screening of patients or high-risk areas for multidrug-resistant bacteria, as well as isolating patients who have been previously colonised or infected during a subsequent admission, are among the strategies that can contribute to reducing the transmission of these bacteria, thereby reducing infections [35].

The percentage of contamination of the hospital environment by *S. aureus* is 13%, including 1.25% MRSA, particularly on wet surfaces, serum racks, bed and bedding. This rate is lower than that found in a northern Algerian hospital (18%) and an Australian hospital (50%) [36,37] but similar to Brazilian studies (12.4% *S. aureus* and 1.7% MRSA) [38]. Various studies have demonstrated that the environment, equipment and utensils used in the clinical setting play a fundamental role in maintaining endemic *S. aureus* during MRSA outbreaks [39]. In our study, the same clones of different origin, especially environmental, were isolated at intervals of several months. This is consistent with the literature, which estimates that *S. aureus* can persist between seven days and seven months on inanimate surfaces [40].

In our study, the dominant SCC*mec*IV accounted for eight out of ten of the isolated MRSAs, while two were SCC*mec*Vc. These types were reported to be associated with the community, in contrast to types I, II and III, which were associated with hospitals [41]. These results are consistent with other studies in China, Brazil and Armenia, which characterised MRSA isolated from hospital settings and patient nasal carriage and found a predominance of CA-MRSA in nosocomial settings, exposing the flexibility of hospital–community boundaries [38,42,43].

In Algeria, the MRSA-ST80 clone is the predominant clone found in nasal carriage, human samples, animals, food, and water [13,44]. It is a dominant international clone in Europe and is increasingly described in the Middle East [45]. We have described other international clones including MRSA-ST22, notably associated with healthcare infections in the United Kingdom [46]. ST30 has been reported in several studies in different countries as a major clonal complex with a significant impact on human health worldwide. One US study described it as having a higher physical condition in bloodstream infections, which may have an impact on its ability to cause embolisms [47,48]. It has also been identified, alongside ST5, as one of the main types associated with community-acquired MRSA infections in Argentina [49]. However, other less well-known clones, such as ST6 and ST398, have also been reported. ST6 has been implicated in infectious transmission in communities and hospitals and ST15 in cystic fibrosis patients in China, Europe and the Czech Republic [50,51]. Concerning ST398, which has rarely been described in Algeria [44], it was initially associated with livestock and subsequently detected in workers in close contact with livestock [52]. An increasing number of serious infections mainly caused by ST398-MSSA strains were reported in a Canadian-Chinese study [52]. As in our case, the presence of clindamycin- and erythromycin-resistant MSSA-ST398 has been observed in the United States, where it is increasing [53].

Therapeutically, one of the few alternatives to combat emerging resistance to methicillin in MRSA is clindamycin. However, the emergence of strains that are resistant to MLSBs present a new challenge in the treatment of staphylococcal infections [54]. The iMLSB profile was observed in 17.4% of the strains. Our results are in agreement with those reported in other studies conducted in India but are higher than studies conducted in Niger and Brazil [55,56]. In our case, this resistance is mainly mediated by the *ermT* gene and no *ermB* was detected. The few studies conducted in Algeria have only detected *ermA*, *ermB*, *ermC* and did not detect *ermT.* Our study seems to show a specificity with a preponderance of the *ermT* gene compared to the *ermB* predominant in many countries [13,44,57,58,59,60]. Clindamycin can also lead to the suppression of virulence factors in these bacteria, where it decreases the production of PVL, TSST and HLA [61].

Toxins such as PVL and TSST-1 generated by *S. aureus* play an essential role in the pathogenesis of the infection [62]. In addition to being responsible for toxic shock syndrome and suppurative infections, TSST-1 with enterotoxins induces T-cell proliferation without antigenic specificity [57]. PVL is associated with necrotising pneumonia and soft tissue infections, playing a mechanistic role in neutrophil lysis [63]. All our ST80-MRSA strains are PVL+, which is typical of European ST80-MRSA isolates, of which 90% are PVL+ [64]. In total, 20% of *S. aureus* have been described in China as producers of TSST-1, often associated with different STs including ST5, ST22, ST6 and ST30 [60,65].

Other virulence factors playing a role in human health, notably associated with staphylococcal food poisoning, have been detected in strains in the hospital environment and in carriers such as the *sea*, *seb* and *seh* enterotoxin genes [58,66,67]. The *icaADBC* and *icaR* genes, responsible for biofilm formation, mucus production and its regulation, and facilitating attachment to environmental surfaces, were found in all isolates, explaining the difficulty of eradicating these strains [64]. The *scn* gene that specifically blocks activation of the human complement system was identified in all our strains [68]. The immune evasion cluster (combination of *chp*, *sak*, *scn* and/or *sea*) specific to humans and permitting adaptation to the human host was present in environmental strains of ST22 [13,69]. These MRSA-IV-ST22-TSST-1+ strains appear to come from a person who contaminated their environment. This supports the hypothesis of a bacterial exchange between nasal carriage and the hospital environment, both of which are, in turn, a reservoir of pathogens.

## 5. Conclusions

This study describes the prevalence and characterisation of *S. aureus* in the nasal carriage and the hospital environment, as well as the circulation of different pathogenic clones of MRSA, MSSA and iMLSB in an Algerian hospital. This research indicates the presence of a mosaic of international clones, including ST22, ST30 and ST80. In addition, the emergence of MSSA-ST398-iMLSB+ and MRSA-IV-ST398-Imlsb + clones should be monitored. The presence of the same pathogenic strains in the hospital environment and in carriers supports their role as a potential reservoir for postoperative infections. To prevent environmental contamination and nosocomial infections, preventive measures such as reinforced hygiene measures for staff and patients, as well as effective and regular disinfection of all equipment and surfaces must be implemented. Systematic screening of patients with risk factors should be implemented if possible. 

## Figures and Tables

**Table 1:** Prevalence of *S. aureus* strains by number of samples, resistance phenotype and origin.

	Number of Samples	*S. aureus*	MSSA	MRSA
**Patients (%)**	110 (20%)	29 (27%)	26 (23.6%)	3 (2.7%)
**HWs (%)**	40 (7.3%)	12 (30%)	10 (25 %)	2 (5%)
**Environment (%)**	400 (72%)	51 (13%)	46 (11.5%)	5 (1.25%)
Total	550	92	82	10

**Table 2:** Distribution of virulence genes on the 22 sequenced strains.

Genes	Environment Origin (*n* = 12)		Patient Origin (*n* = 7)		Health Worker Origin (*n* = 3)	
	MSSA (*n* = 7)	MRSA (*n* = 5)	MSSA (*n* = 4)	MRSA (*n* = 3)	MSSA (*n* = 1)	MRSA (*n* = 2)
			**Toxins**			
***sea*** ***seb*** ***seh*** ***tst*** ***lukf-pv*** ***luks-pv***	2 (29%) 0 (0%) 0 (0%) 1 (14%) 0 (0%) 0 (0%)	1 (20%) 1 (20%) 1 (20%) 2 (40%) 3 (60%) 1 (20%)	0 (0%) 0 (0%) 0 (0%) 0 (0%) 1 (25%) 0 (0%)	2 (66%) 0 (0%) 1 (33%) 0 (0%) 3 (100%) 1 (33%)	0 (0%) 0 (0%) 0 (0%) 0 (0%) 1 (100%) 0 (0%)	0 (0%) 1 (50%) 0 (0%) 0 (0%) 1 (50%) 0 (0%)
**Haemolysins**						
***hla*** ***hlb*** ***hld*** ***hlgA*** ***hlgB*** ***hlgC***	7 (100%) 7 (100%) 7 (100%) 7 (100%) 7 (100%) 7 (100%)	5 (100%) 5 (100%) 5 (100%) 5 (100%) 5 (100%) 5 (100%)	4 (100%) 4 (100%) 4 (100%) 4 (100%) 4 (100%) 4 (100%)	3 (100%) 3 (100%) 3 (100%) 3 (100%) 3 (100%) 3 (100%)	1 (100%) 1 (100%) 1 (100%) 1 (100%) 1 (100%) 1 (100%)	2 (100%) 2 (100%) 2 (100%) 2 (100%) 2 (100%) 2 (100%)
**MSCRAMMs (Adhesins)**						
***cna*** ***ebp*** ***clfA*** ***clfB*** ***fnbA*** ***fnbB***	2 (29%) 3 (43%) 7 (100%) 7 (100%) 5 (71%) 5 (71%)	4 (80%) 3 (60%) 5 (100%) 5 (100%) 5 (100%) 5 (100%)	0 (0%) 1 (25%) 4 (100%) 2 (50%) 4 (100%) 4 (100%)	2 (66%) 3 (100%) 2 (66%) 3 (100%) 3 (100%) 3 (100%)	0 (0%) 1 (100%) 1 (100%) 1 (100%) 1 (100%) 1 (100%)	0 (0%) 1 (50%) 2 (100%) 2 (100%) 2 (100%) 2 (100%)
**Capsule components**						
***cap8*** ***icaA*** ***icaB*** ***icaC*** ***icaD*** ***icaR***	1 (14%) 7 (100%) 7 (100%) 7 (100%) 7 (100%) 7 (100%)	3 (60%) 5 (100%) 5 (100%) 5 (100%) 5 (100%) 5 (100%)	1 (25%) 4 (100%) 4 (100%) 4 (100%) 4 (100%) 4 (100%)	3 (100%) 3 (100%) 3 (100%) 3 (100%) 3 (100%) 3 (100%)	1 (100%) 1 (100%) 1 (100%) 1 (100%) 1 (100%) 1 (100%)	0 (0%) 2 (100%) 2 (100%) 2 (100%) 2 (100%) 2 (100%)
**Other factors**						
***scn*** ***chp*** ***sak***	7 (100%) 5 (71%) 1 (14%)	5 (100%) 2 (40%) 5 (100%)	4 (100%) 4 (100%) 0 (0%)	3 (100%) 0 (0%) 3 (100%)	1 (100%) 1 (100%) 0 (0%)	2 (100%) 1 (50%) 2 (100%)

MSCRAMMs: Microbial Surface Components Recognising Adhesive Matrix Molecule.

## Data Availability

The data presented in this study are openly available in NCBI in Bioproject PRJNA836883.

## Supplementary Materials

The following are available online at https://www.mdpi.com/article/10.3390/antibiotics11070971/s1, Table S1: Detail of the distribution of environmental samples according to the isolation site and the presence of *S. aureus*, MSSA and MRSA, Table S2: Details of healthcare worker sample distribution according to their role and the presence of *S. aureus*, MSSA and MRSA, Table S3: Details of patient sample distribution according to age and gender of patients and by the department in which they were hospitalised and the presence of *S. aureus*, MSSA and MRSA.
