# Supplementary material for: Genetic Diversity and Virulence Profile of Methicillin and Inducible Clindamycin-Resistant Staphylococcus aureus Isolates in Western Algeria
Source: Antibiotics (Basel). 2022 Jul 19;11(7):971. doi: 10.3390/antibiotics11070971 (PMC9312111; doi:10.3390/antibiotics11070971)
Supplement: Supplementary file 1 [file antibiotics-11-00971-s001.zip › antibiotics-1797834-supplementary.pdf]

Supplementary Table S1. Detail of the distribution of environmental samples according to the isolation site and the presence of *S. aureus,* MSSA and MRSA.

	Isolation site	Number of samples	*S. aureus*	MSSA	MRSA
**Surface areas**	Wet surfaces	32	8	7	1
	Patient's Beds	30	6	5	1
	Serum racks	20	5	4	1
	Surgical carts	30	5	5	0
	Door handles	30	4	4	0
	Waiting room benches	42	4	3	1
	Floor ICU	13	1	1	0
	Switches	20	1	1	0
	Trash can lid	30	1	1	0
	Surgical cart tray	10	6	6	0
	Surgical scissors and clips	13	1	1	0
	Doctor's office	5	0	0	0
	Surgical tables	11	0	0	0
**Biomedical equipment**	Respirators ICU	13	2	1	1
	Laryngoscope	30	2	2	0
	Redon drains	30	2	2	0
	Urinary catheter bags	20	2	2	0
	Block scope	16	1	1	0
	ICU scope	5	0	0	0
**Total**		**400**	**51**	**46**	**5**

ICU: Intensive Care Unit.

Supplementary Table S2. Details of healthcare worker sample distribution according to their role and the presence of *S. aureus*, MSSA and MRSA.

Function	Date of collection	Number of samples	*S. aureus*	MSSA	MRSA
**General surgeon**	14.03.21	7	3	2	1
**Orthopedic surgeon**		5	0	0	0
**Chief of the block**		1	1	1	0
**Anesthetist**		3	1	1	0
**Nurse**		13	4	3	1
**Caregiver**		8	3	3	0
**Coordinator**		1	0	0	0
**Cleaning agent**		2	0	0	0
**Total**		**40**	**12**	**10**	**2**

Supplementary Table S3. Details of patient sample distribution according to age and gender of patients and by the department in which they were hospitalised and the presence of *S. aureus*, MSSA and MRSA.

Date of collection	Total of samples	Gender		Age				Number of samples		*S. aureus*		MSSA		MRSA	
		M	F	0-14 years	15-24 years	25-64 years	65 and more	G	O	G	O	G	O	G	O
**08.11.20**	3	1	2	0	0	2	1	3	0	1	0	1	0	0	0
**10.11.20**	6	1	5	0	2	4	0	4	2	2	0	2	0	0	0
**15.11.20**	2	0	2	0	0	2	0	2	0	0	0	0	0	0	0
**18.11.20**	5	2	3	1	0	3	1	3	2	2	0	2	0	0	0
**25.11.20**	10	8	2	0	3	5	2	7	3	1	1	1	1	0	0
**02.12.20**	13	6	7	0	1	8	4	8	5	3	2	3	2	0	0
**06.12.20**	5	3	2	0	0	4	1	5	0	2	0	1	0	1	0
**09.12.20**	10	4	6	0	1	6	3	3	7	1	4	1	4	0	0

**10.01.21**	9	2	7	0	3	5	1	8	1	2	1	2	1	0	0
**13.01.21**	8	3	5	0	1	7	0	5	3	0	0	0	0	0	0
**24.01.21**	10	8	2	2	0	8	0	8	2	2	0	1	0	1	0
**27.01.21**	9	5	4	0	1	6	2	5	4	1	0	1	0	0	0
**31.01.21**	8	6	2	0	2	5	1	6	2	0	1	0	1	0	0
**23.03.21**	5	3	2	0	0	5	0	3	2	0	2	0	2	0	0
**28.03.21**	7	0	7	0	2	3	2	4	3	1	0	0	0	1	0
**Total**	**110**	**52**	**58**	**3**	**16**	**73**	**18**	**74**	**36**	**18**	**11**	**15**	**11**	**3**	**0**

M: Male, F: Female, G: General surgery department, O: Orthopaedic surgery department.
